# Ultra Low-Coverage Whole-Genome Sequencing as an Alternative to Genotyping Arrays in Genome-Wide Association Studies

**DOI:** 10.3389/fgene.2021.790445

**Published:** 2022-02-15

**Authors:** Vylyny Chat, Robert Ferguson, Leah Morales, Tomas Kirchhoff

**Affiliations:** ^1^ Perlmutter Cancer Center, New York University School of Medicine, New York, NY, United States; ^2^ Departments of Population Health and Environmental Medicine, New York University School of Medicine, New York, NY, United States; ^3^ The Interdisciplinary Melanoma Cooperative Group, New York University School of Medicine, New York, NY, United States

**Keywords:** ultra low-coverage whole-genome sequencing (ulcWGS), genome-wide association study (GWAS), next-generation sequencing (NGS), illumina global screening array, genomic assay comparison

## Abstract

An array-based genotyping approach has been the standard practice for genome-wide association studies (GWASs); however, as sequencing costs plummet over the past years, ultra low-coverage whole-genome sequencing (ulcWGS <0.5× coverage) has emerged as a promising alternative that provides superior genomic coverage with substantial reduction of genotyping cost. To evaluate the potential utility of ulcWGS, we performed a whole-genome sequencing (WGS) of 72 European individuals to a target coverage of 0.4× and compared its performance with the widely used Infinium Global Screening Multi-Disease Array (GSA-MD). We showed that the number of variants captured by ulcWGS is comparable with imputed GSA-MD platform, particularly for low-frequency (95.5%) and common variants (99.9%), with high imputation R^2^ accuracy (mean 0.93 for SNPs and 0.86 for indels). Using deep-coverage 30× WGS as the “truth” genotypes, we found that ulcWGS has higher overall nonreference genotype concordance compared with imputed GSA-MD for both SNPs (0.90 vs. 0.88) and indels (0.86 vs. 0.83). In addition, ulcWGS proved to be as sensitive as the genotyping-based method in sex imputation and ancestry prediction producing similar principal component (PC) scores. Our findings provide important evidence that the cost efficient ulcWGS of <0.5× generates high genotype accuracy, outperforming the standard genotyping arrays, making it an attractive alternative to the array-based method in next-generation GWAS design.

## Introduction

Over the past decade, genome-wide association studies (GWASs) have identified genetic variation contributing to a plethora of complex disease traits (Visscher et al., 2012; Visscher et al., 2017; Buniello et al., 2019). GWASs have routinely used dense genotyping arrays to assay fixed panels of hundreds of thousands to millions of common genetic markers, followed by imputation through population reference panels, to increase the density of the studied genetic variation coverage (Visscher et al., 2017). Despite the high genotype accuracy and affordable cost, genotype-based GWAS designs are often limited by ascertainment bias of the genotyped variants present at particular SNP arrays. This limits the genome-wide variant coverage particularly for the discovery of the association with novel and/or rare variants. Recently introduced whole-genome sequencing (WGS)-based GWAS emerges as a promising alternative to probe large fraction of genetic variation in a comprehensive and unbiased fashion, improving the power of the association tests and downstream fine mapping analyses. However, high-coverage WGS remains cost prohibitive in GWAS designs that often require assessment of a large cohort of sample population.

To address these limitations, low-coverage WGS (lcWGS) has recently been proposed as an attractive cost-efficient technological approach, sequencing random genomic regions at a reduced sequencing coverage. Studies show that lcWGS significantly supersedes SNP arrays on variant density, which also allows for a more thorough assessment of associations with less common variants. Leveraging on haplotype information from fully sequenced reference panels [i.e., 1000 genome project (1 KGP) or the haplotype reference consortium (HRC)], lcWGS sequencing outputs are further improved by robust imputation tools to increase resolution, unconstrained by fixed genotyping array probes. Several GWAS studies have successfully employed lcWGS-based designs with as low as 1.5× sequencing coverage, in trait-associated variant discovery (consortium, 2015; Tachmazidou et al., 2017; Luo et al., 2017; consortium et al., 2015). However, even with 1.5× depth compared with the SNP array genotyping, the cost is still too high, prohibitive for the large population screening, particularly in multinational consortia setting. Ultra low-coverage WGS (ulcWGS ≤0.5× sequencing depth) would represent additional cost reduction, substantially below SNP array genotyping; however, it has not yet been extensively evaluated for its potential as a sequencing-based GWAS alternative.

A few prior reports assessing the accuracy and efficiency of ultra low-coverage WGS (0.1–0.5× ulcWGS) were limited to *in silico* simulation or downsampling of high-quality deep-coverage WGS data (Pasaniuc et al., 2012; Gilly et al., 2019; Homburger et al., 2019; Wasik et al., 2021). While promising, many of these studies did not account for all aspects of sequencing library preparation needed for such low coverage or reduced DNA inputs that might each have impacted ulcWGS experimental designs and could have resulted in biased estimates of ulcWGS performance. While studies that modeled low coverage from high-depth sequencing data suggested the potential utility of ulcWGS in GWAS designs at sequencing coverage as low as 0.1× (Pasaniuc et al., 2012), up to date, there has only been one study that used low-coverage sequencing design, at 0.5× depth, to evaluate the performance of ulcWGS platform (Li et al., 2021). In our study, we performed low-coverage sequencing to less than 0.5× depth, making this ulcWGS technological platform even more cost competitive. To investigate the effectiveness of ulcWGS as a viable alternative to genotyping arrays, we used the imputed data from the Global Screening Multi-Disease Array (GSA-MD) as the genotype gold standard in our primary analysis. For a subset of samples, we further compared and assessed the performance and accuracy of genotyping-based vs. ulcWGS data with high-coverage (30×) WGS.

## Methods

### Study design and sample selection

The study population was derived from a melanoma patient cohort ascertained at New York University Langone Health (NYULH). We selected 72 samples with high-quality genotyping data previously generated using the Infinium Global Screening Array Multi-disease drop-in (GSA-MD V2.0 and V3.0) and available sequencing data from high-coverage whole-genome sequencing (30× hcWGS). To test the effectiveness of ulcWGS as a cost-efficient alternative to genotyping-based GWAS, we sequenced all 72 individuals to a target 0.4× sequencing coverage (lower per-sample cost compared with GSA-MD V3.0). All study participants were of self-reported European descent. Written informed consents for the use of specimens were obtained at the time of enrollment, and the Institutional Review Board (IRB) at NYULH approved the study.

### Ultra low-coverage whole-genome sequencing: data generation and QC

We isolated genomic DNA from whole-blood samples provided by study participants using Qiagen DNEASY blood and tissue kit. Qubit was used to quantify DNA concentration, and the DNA quality was assessed by gel electrophoresis. All 72 DNA samples selected in this study had a minimum concentration of 10 ng/µl with no evidence of DNA degradation from gel electrophoresis. Libraries were prepared by GENEWIZ using the NEBNext® Ultra™ II DNA Library Prep Kit for Illumina following the recommendations of the manufacturer, including eight PCR cycles. The libraries were quantified using real-time PCR, clustered on two lanes of a flow cell and loaded on an Illumina HiSeq instrument following the instructions of the manufacturer. The samples were sequenced using a 2 × 150-bp paired-end configuration. Raw sequencing read yields ranged from 1,300 to 2,623 Mb (mean 1,623 Mb, approximately 0.5× coverage). The raw sequencing outputs were in FASTQ format.

### ulcWGS: sequencing alignment, genotype calling and imputation-based genotype refinement

Sequencing FASTQ files were aligned to Homo_sapiens_assembly19. fasta (GRCh37) from GATK bundle using Bwa-mem 0.7.17, following GATK best practices. Sambamba-0.6.8 markdup was used to mark duplicate reads and BaseRecalibrator function from GATK v. 4.1.2.0 to perform base quality score recalibration (BQSR). We calculated mean depth coverage excluding nonprimary alignment, unmapped and duplicate reads from the processed alignment files (BAM files) with Mosdepth v.0.2.6.

We estimated genotype likelihoods (GL) of the processed bam files for all variable genomic positions in the 1 KGP phase 3 reference panel (GRCh37) with bcftools v.1.9 mpileup, followed by left-aligned normalization of indels and splitting of multi-allelic variants into biallelic SNP formats. Subsequently, we refined and imputed the raw GL estimates of autosomal chromosomes and non-pseudoautosomal (non-PAR) region of chromosome X with GLIMPSE 1.0.0, a newly developed software suite tailored toward efficient and accurate imputation-based refinement for ulcWGS (Rubinacci et al., 2021). GLIMPSE utilized Gibbs sampling procedure to iteratively refine the initial GL estimates conditioning on haplotypes from the input reference panel and other target samples in each iteration. We followed the protocols of GLIMPSE’s, with slight modifications to include indels and SNPs, accessible at https://odelaneau.github.io/GLIMPSE/tutorial_hg19.html and https://odelaneau.github.io/GLIMPSE/tutorial_chrX.html, for autosomal and non-PAR X chromosome, respectively.

### Infinium Global Screening Array Multi-Disease: genotype calling and imputation

All 72 DNA samples in the study were genotyped using GSA-MD V2.0/V3.0 that included additional ∼50,000 genomic markers as a multi-disease drop-in. We used Illumina Genome Studio V2.0 to generate genotype calls from raw intensity idat files with GenCall score threshold of 0.15. PLINK Input Report Plug-in v2.1.4 exported the genotype calls from Genome Studio to PLINK format for downstream analyses.

GSA genotype data were imputed using open-source Michigan Imputation Server using the 1 KGP phase 3 GRCh37 reference haplotypes (Das et al., 2016), generating imputation dosage files along with log files and imputation info score reports.

### High-coverage whole-genome sequencing: data generation and processing

Among 72 samples sequenced by ulcWGS and genotyped by GSA-MD V2/V3, 13 samples had available high-coverage WGS data (30×) generated previously. The raw FASTQ files were processed, and variants were called using GATK variant calling best practice workflow, in which we used HaplotypeCaller to simultaneously call SNPs and indels with *de novo* haplotype assembly. We performed a liftover (Picard LiftoverVcf) for the hcWGS vcf from GRCh38 to GRCh37 to be consistent with ulcWGS and GSA-MD data.

### Evaluation of ultra low-coverage whole-genome sequencing performance

To evaluate the performance of ulcWGS capturing the variation identified by the imputed GSA-MD platform, we compared the variant data from ulcWGS and imputed GSA-MD of autosomal chromosomes to identify 1) overlapping variants discovered by both platforms and 2) unique variants identified in each platform. As a secondary analysis, we utilized 30X hcWGS data generated on a subset of 13 participants from our study cohort and assessed the ability of ulcWGS vs. imputed GSA-MD in recapitulating 30X hcWGS non-monomorphic variants at different population minor allele frequencies (MAFs).

To assess genotype accuracy of ulcWGS, we first compared the imputation mean R^2^ scores of ulcWGS with those of imputed GSA-MD stratified by population MAF (50 MAF bins: MAF <1, 1–2,2–3, … ,49–50). Imputation R^2^, which measures correlation between empirical allelic dosage and expected true genotype, ranges from 0 to 1 with a higher score suggesting higher imputation certainty (Marchini and Howie, 2010). Subsequently, as an additional metrics for the assessment of genotype accuracy of ulcWGS, we calculated non-reference concordance (NRC) by excluding homozygote reference concordance. As described in detail elsewhere (Martin et al., 2021), such reference concordance exclusion reduced overestimated concordance, in particular, for rare variants. We filtered imputed GSA-MD data at multiple imputation R^2^ thresholds (>0.3, >0.5, >0.6, >0.8, and >0.9) and used each as gold standard. Nonreference concordance between the gold standard and unfiltered ulcWGS was computed separately for indels and SNPs. As a supplemental analysis, we also calculated NRC of ulcWGS and GSA-MD, both filtered at each corresponding R^2^ thresholds. To further characterize ulcWGS accuracy, we capitalized on the data from 30× hcWGS available for a subset of 13 samples, and used this data as the “gold standard” genotypes. We calculated non-reference concordance for unfiltered ulcWGS and imputed GSA-MD against 30× hcWGS to compare genotype accuracy of the ulcWGS and genotyping-based GSA-MD platforms.

We have also explored the potential utility of ulcWGS for sex imputation; as to our knowledge, this has never been tested in low-pass WGS analyses. With novel GLIMPSE pipeline to impute the non-PAR X chromosome from ulcWGS, we obtained raw genotype calls, which we further filtered to retain high-quality variants (imputation R^2^ >0.5; PLINK hardcall >0.8; missing rate <20%). Similarly, for GSA-MD chip data, we performed a less conservative filtering, in which variants and samples of missing rate >20% were removed, as per recommendations from GWAS QC pipelines (Marees et al., 2018). Subsequently, based on heterozygosity rate of non-PAR X chromosome, we imputed sex information with PLINK 1.9 using the default threshold of F inbreeding coefficient estimates (F <0.2: females and F >0.8: males). We compared the concordance of sex imputed from ulcWGS vs. GSA-MD against reported sex information.

As population structure is a common confounding factor in GWAS, we assessed if sequencing-based ulcWGS data output can stratify ancestry with comparable sensitivity performed by genotyping-based GSA-MD array. Using PLINK 2.0 (Chang et al., 2015), we performed variant QCs under the following variant exclusion criteria: genotypes with posterior probability <0.8 (PLINK hardcall flag >0.8); variants with imputation R^2^ <0.5, variants with call rate <95%, or those deviated from the Hardy–Weinberg equilibrium HWE p <5E−07. Variants passing filtering criteria from both ulcWGS and GSA-MD chip data were merged. To the merged file, we further added genotypes from the 1 KGP phase3, to provide ancestry reference for our study. Sex chromosomes, rare alleles (maf <0.05), ambiguous variants, and variants with missing rate >5% were filtered out. We performed LD pruning using standard parameters: --indep-pairwise 500 10 0 pruning prior to running --pca. Pairwise kinship coefficients measuring distance and quantifying similarity among samples were also estimated using PLINK 2. 0 --make-kin-table for a subset of pruned variants used in PCA. We expected the same sample generated by ulcWGS and GSA-MD to have kinship coefficients ranging from 0.35 to 0.5, which would suggest strong similarity regardless of the platform. To further characterize discrepancy in raw PC scores generated by these two platforms that may affect multivariable model adjustment in downstream GWAS analyses, we estimated Pearson R^2^ correlation of the top 10 PC scores of the same individual from ulcWGS and GSA-MD chip. For a more in-depth refinement of European ancestry substructure, we have used the data from European population 1 KG phase 3 and reference Ashkenazi Jewish (AJ) population data previously published by our group (Vijai et al., 2013; Ferguson et al., 2019). We added AJ population into the ancestry reference since a fraction of the NYULH patient cohort in this study is of AJ ancestry.

## Results

**FIGURE 1:**
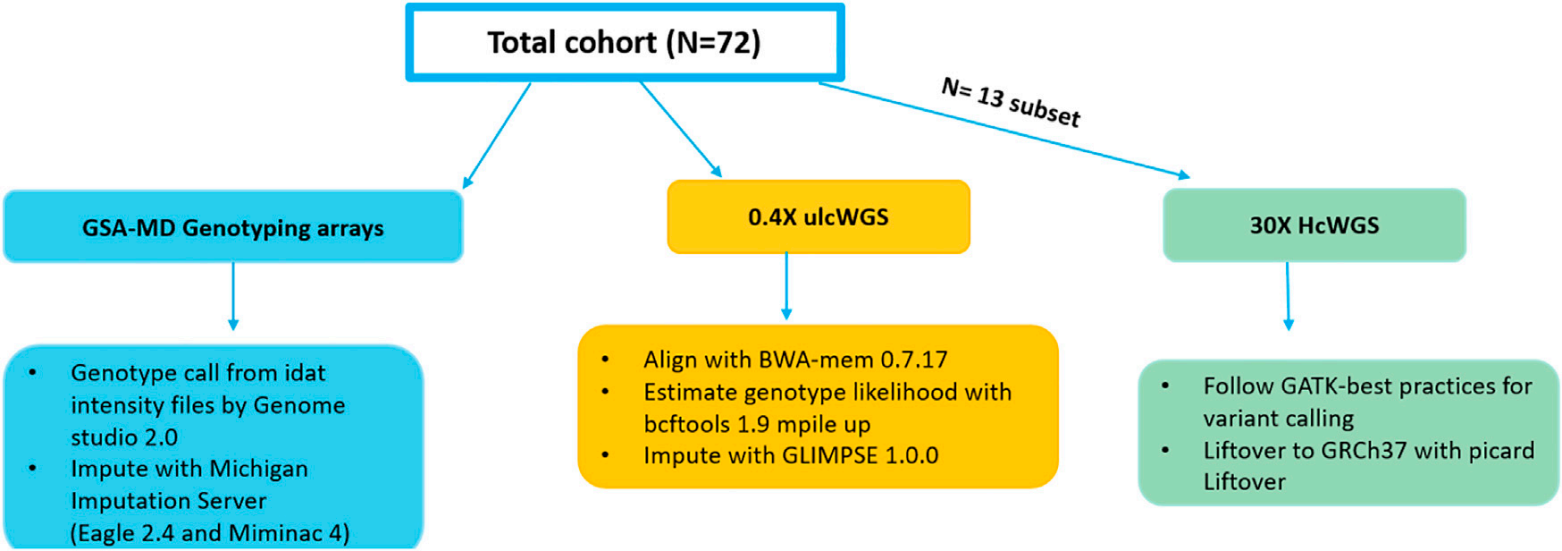
Schematic workflows of the study design and analytical pipelines. The total 72 individuals of European descent in the study were genotyped with Infinium Global Screening Multi-Disease Array (GSA-MD) (V2.0/V3.0) and whole-genome sequenced to a target 0.4× coverage. A subset of 13 samples had high-coverage whole-genome sequencing (WGS) (30X) data available and were used to conduct a secondary analysis to assess ultra low-coverage whole-genome sequencing (ulcWGS) performances against genotyping-based GSA-MD.

In this study, we have tested the ability of ulcWGS (0.4×) to be used as a feasible alternative to genotyping-based arrays. On a sample of 72 melanoma patients with genotype data generated by GSA-MD (v2 or v3), we performed ulcWGS to a target depth of 0.4× sequencing coverage (Figure 1). GSA-MD genotype data were imputed using Michigan imputation server utilizing 1 KGP phase 3 build GRCh37 as a reference panel. UlcWGS data were processed to generate genotype likelihood for all polymorphic sites using bcftools, prior to imputation with Glimpse v1. To further evaluate ulcWGS performance, our study also included 30× hcWGS of 13 individuals (stemming from 72 sample cohort), which were processed following GATK variant calling pipelines using build GRCh37. Mean sequencing coverage for ulcWGS data from raw and post-alignment processing were 0.49× and 0.38×, respectively (Supplementary Figure S1).

The raw and imputed fraction of detected non-monomorphic variants from all three platforms is summarized in Table 1. There were 759,993 and 730,059 variants directly typed onto the GSA-MD arrays v2 and v3, respectively. We merged 41 samples genotyped on GSA-MD v2 with 31 samples processed with GSA-MD v3 and performed imputation, which resulted in a panel of 11,430,960 non-monomorphic variants (10,157,732 SNPs and 1,264,727 indels). UlcWGS on all 72 samples identified 11,673,829 non-monomorphic variants (10,384,933 SNPs and 1,280,217 indels), which is comparable with imputed data from GSA-MD analysis. We also further applied multiple imputation R^2^ filtering thresholds (>0.3, >0.5, >0.6, >0.8, and 0.9) to both imputed GSA-MD and ulcWGS data, and consistently observed a comparable number of variants from these two platforms at respective R^2^ thresholds (Supplementary Table S1). For 30× hcWGS data, the number of nonmonomorphic variants was smaller (*N* = 9,737,221) in comparison with imputed GSA-MD and ulcWGS (Table 1), reflecting a reduced cohort size (*N* = 13).

**TABLE 1:** Non-monomorphic variants captured by different platforms in this study.

Variants	Infinium Global Screening Multi-Disease Array (GSA-MD) (*N* = 72)	0.4X ultra low-coverage whole-genome sequencing (ulcWGS) (*N* = 72)	30X hcWGS (*N* = 13)
Raw number of variants	759,993 (v2.0)	NA^*^	9,737,221 (nonmonomorphic)
	730,059 (v3.0)		
Imputed number (nonmonomorphic)	11,430,960	11,673,829	9,737,221
Number of SNPs	10,157,732	10,384,933	7,351,799
Number of indels	1,264,727	1,280,217	2,240,753

Note. All variants post-imputation for GSA-MD and ulcWGS platforms were unfiltered, with the exception of monomorphic variants. Similarly, 30× hcWGS data generated by GATK pipelines included only nonmonomorphic variants. The number of SNPs and indels were estimated separately. NA*: The pipeline to process ulcWGS data did not generate raw genotype hard calls; instead, it calculated posterior genotype likelihood for all polymorphic sites in the reference panel, later refined by the imputation tool GLIMPSE 1.0.0.

**FIGURE 2:**
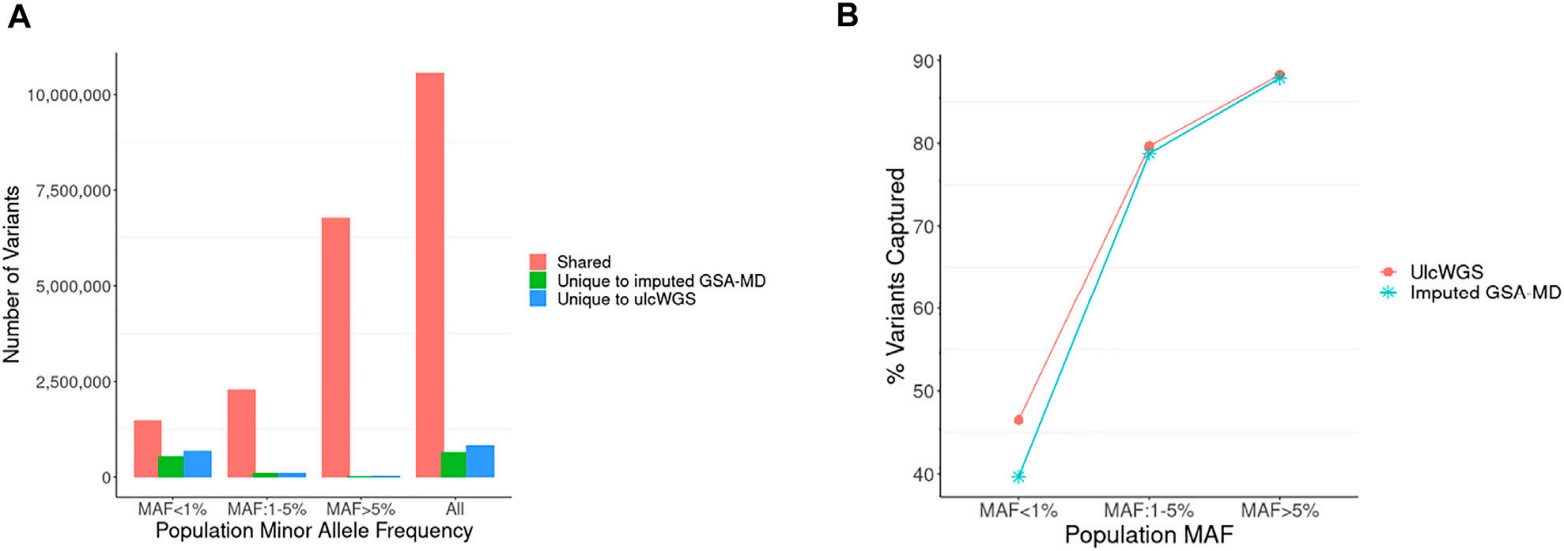
Comparison of variant fraction discovered by ulcWGS vs. imputed GSA-MD. **(A)** Imputed GSA-MD and ulcWGS data (*N* = 72) were filtered to retain only nonmonomorphic variants. We compared the filtered variants between both platforms to evaluate the comprehensiveness of ulcWGS in capturing variants generated by imputed GSA-MD. Numbers of shared and unique variants to each platform were plotted by population MAF derived from non-Finland European population in gnomAD v2.0. **(B)** The fraction of non-monomorphic variants (Y-axis) from 30× hcWGS captured by ulcWGS vs. imputed GSA-MD (*N* = 13 participants genotyped on all three platforms) by different population MAF bins (X-axis). At low (1–5%) and common (>5%) population MAF, both platforms performed similarly. For rare variants (MAF <1%), ulcWGS recapitulated higher fraction of 30X hcWGS variants, as shown by more pronounced separation of ulcWGS (red line) and imputed GSA-MD (blue line).

To assess the efficiency of ulcWGS in capturing genetic variation genotyped by GSA-MD arrays, we compared nonmonomorphic variants generated by these two platforms (Figure 2). UlcWGS captured a vast majority of variants from imputed GSA-MD (94.2%), particularly for common variant MAF >5% (99.9%) and low-frequency variants MAF: 1–5% (95.5%). For rare variants (MAF <1), imputed GSA-MD generated 542,846 variants unique to GSA-MD, while ulcWGS generated 682,801 variants, capturing ∼25% extra rare/novel variants. As a secondary analysis, we also evaluated how comprehensive ulcWGS vs. imputed GSA-MD are in capturing nonmonomorphic variants generated by 30× hcWGS (N subset = 13). As expected, we observed a comparable fraction of 30× hcWGS variants recapitulated by these two platforms at common (MAF > 5%) (∼88% for both platforms) and low frequency (MAF 1–5%) (∼79% for both platforms) (Figure 2B and Supplementary Table S2). For rare variants (MAF <1%), ulcWGS captured noticeably higher fraction of 30X hcWGS variants compared with imputed GSA-MD (46.48% vs. 39.64%) (Supplementary Table S2).

**FIGURE 3:**
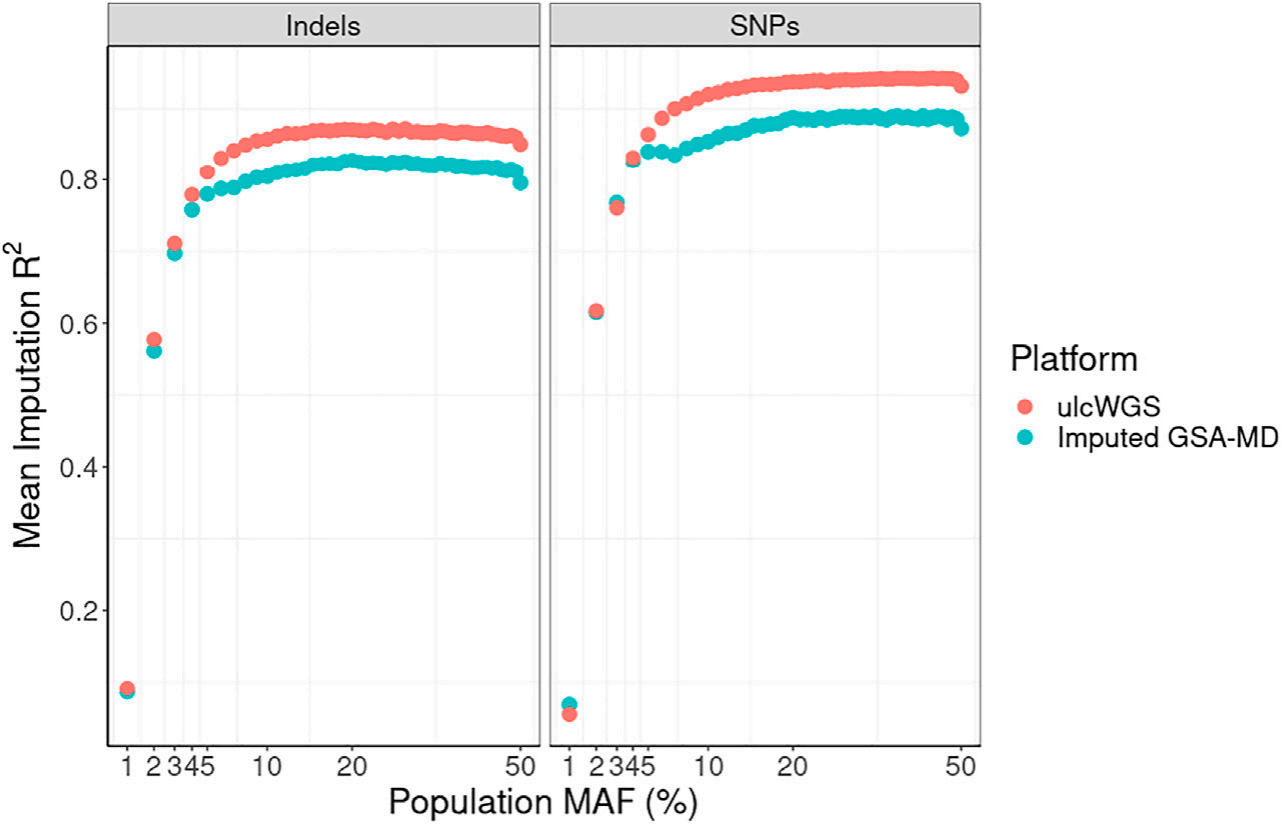
Imputation R^2^ score by population minor allele frequency. Mean imputation R^2^ values were calculated separately for indels and SNPs and stratified by population minor allele frequency (European gnomAD v2.0). All variants were unfiltered.

To evaluate the quality of the input genotype data (ulcWGS vs GSA-MD), we estimated imputation R^2^ score measuring imputation certainty of each imputed variant, using the same reference panel (1 KGP phase 3). Overall, as shown in Figure 3, we observed higher imputation R^2^ scores for ulcWGS platform compared with imputed GSA-MD for both indels and SNPs. We noted that imputation was more accurate in ulcWGS for common variants (MAF >5%) with mean imputation R^2^ of 0.93 for SNPs (vs. 0.87 in imputed GSA-MD) and 0.86 for indels (vs. 0.81 in imputed GSA-MD) (Supplementary Table S3).

**FIGURE 4:**
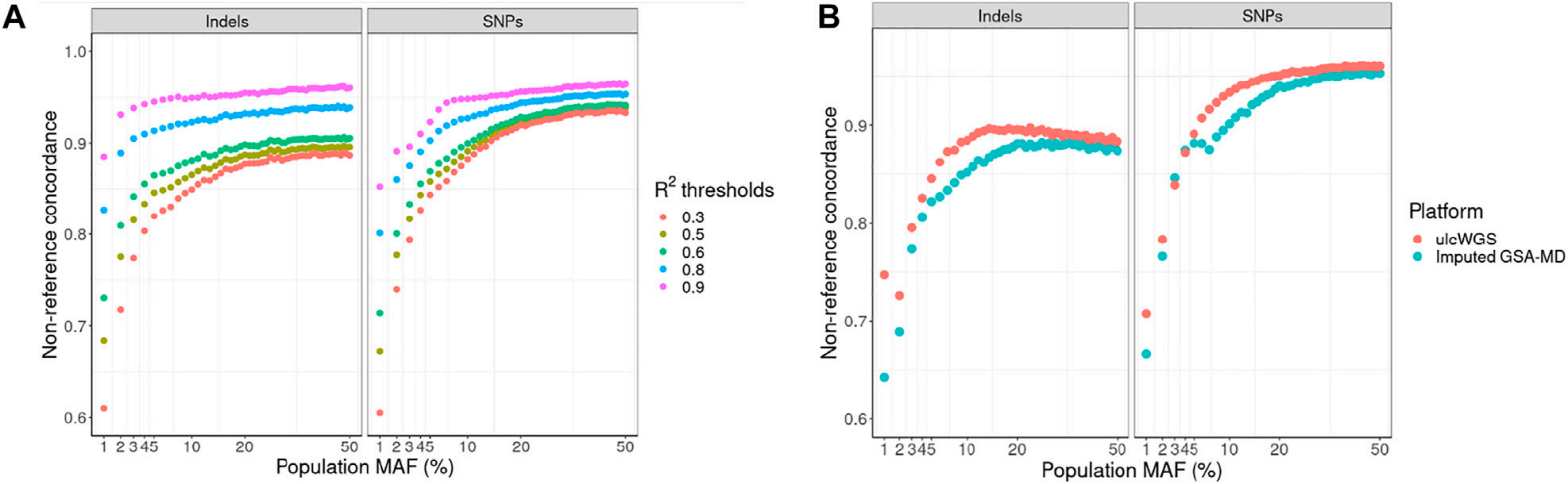
Nonreference concordance by population allele frequency. **(A)** We used imputed GSA-MD variants filtered at different imputation R^2^ thresholds as the “truth” genotypes (R^2^: >0.3, >0.5, >0.6, >0.8, and >0.9). Nonreference concordance was computed for ulcWGS data at various population MAF bins (MAF derived from gnomAD v2.0). To further compare the accuracy of ulcWGS vs. imputed GSA-MD data, we estimated nonreference concordance using 30× hcWGS as the “truth” genotypes **(B)**.

In our primary analysis, we performed quality control of the imputed GSA-MD data at different imputation R^2^ thresholds (0.3, 0.5, 0.6, 0.8, and 0.9) and used these variants as the “truth” genotypes. With increasing filtering R^2^ threshold, for both indels and SNPs, we observed an increase in genotype concordance between unfiltered ulcWGS and imputed GSA-MD across all population MAF bins (Figure 4A). While imputation R^2^ filtering threshold for imputed genotype-based data in GWAS is usually arbitrary, in this analysis, we showed that even with R^2^ threshold as low as 0.3 (default criteria of Michigan imputation server) to include more variants, ulcWGS data still provided appreciable genotype concordance compared with GSA-MD (mean non-ref concordance: 0.82 for indels and SNPs) (Supplementary Table S4). At higher R^2^ thresholds (>0.8), we observed improved mean NRC for SNPs and indels (NRC >0.90). We also estimated NRC with ulcWGS and imputed GSA-MD data, both filtered at multiple imputation R^2^ thresholds, and observed slightly higher NRC between these two platforms (Supplementary Table S5).

Subsequently, we took advantage of 30× hcWGS data available for a subset of 13 samples, to compare nonreference concordance of ulcWGS and imputed GSA-MD against hcWGS. For both indels and SNPs, we observed a higher concordance for ulcWGS compared with imputed GSA-MD (Figure 4B). The overall nonreference concordance for GSA-MD and ulcWGS SNPs was slightly higher than that of indels (SNPs: 0.88 and 0.90; indels: 0.83 and 0.86, respectively) (Supplementary Table S6).

As a sensitivity analysis, we evaluated ulcWGS performances for a specific group of curated variants with high clinical significance (51,334 clinvar and 81,403 exonic variants) directly genotyped on GSA-MD. Many of these variants were rare and not captured in the 1 KGP reference used in ulcWGS imputation. We were able to extract 25,609 clinvar and 52,251 exonic variants from the ulcWGS platform and compared the imputation accuracy and genotype concordance against the GSA-MD. In comparisons with the performance of ulcWGS of all variants, we observed similar imputation R^2^ accuracy and nonreference concordance, particularly for low-frequency and common variant MAF ≥1%, suggesting ulcWGS was comparably efficient in capturing clinically significant variants crucial for GWAS application (Supplementary Figure S2).

To assess the ability of ulcWGS in accurately imputing sex, we computed the X chromosome inbreeding F estimates for both ulcWGS and GSA-MD chip data. GSA-MD assigned *N* = 30 of the study participants as females and *N* = 41 as males, consistent with the reported sex information. While ulcWGS was not able to determine sex information for one sample (F = 0.37; Supplementary Figure S3), it accurately imputed sex for 71 samples (∼99%) (Table 2).

**TABLE 2:** Contingency table of sex imputation from ulcWGS and GSA-MD chip/reported sex.

		ulcWGS		
		**Female**	**Male**	**Unassigned**
GSA-MD/reported sex	Female	30	0	0
	Male	0	41	1
	Unassigned	0	0	0

Note. The diagonal cells represent concordance in sex imputation between ulcWGS and GSA-MD/reported sex. While ulcWGS was not able to impute sex for one male sample, this platform was able to correctly impute sex information for all other study populations (∼99%).

**FIGURE 5:**
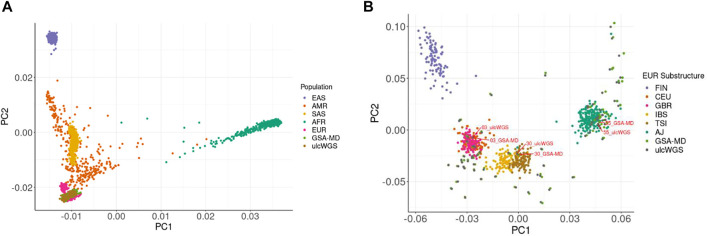
Population stratification of the study cohort using ulcWGS and GSA-MD chip data. We plotted top 2 PCs of the 72 study individuals on top of the 1 KGP phase 3 population **(A)**. The study population clustered and overlapped with the European ancestries from 1 KGP. In **(B)**, we added AJ population to the reference panel of EUR 1 KGP phase 3 and performed PCA to further refine the EUR population substructure. Both ulcWGS and GSA-MD chip similarly captured distinct EUR substructures of the study participants, as exampled by the three labeled sample duplicates overlaid on top of the CEU (sample 03), TSI (sample 30), and AJ (sample 55) subclusters. FIN, Finnish in Finland; CEU, Northern Europeans in Utah; GBR, British in England/Scotland; IBS, Iberians in Spain; TSI, Tuscans in Italy; AJ, Ashkenazi Jewish reference; GSA-MD, based on 72 study participants in this study; ulcWGS, based on 72 study participants in this study.

We computed top principal component scores (PC scores) of the 72 individuals generated by both GSA-MD chip and ulcWGS platforms and performed principal component analysis with 1 KGP phase 3 samples as a population reference (Figure 5A). As expected, for both GSA-MD and ulcWGS platforms, we observed a similar population distribution, clustering within European ancestries, confirming self-reported European descent of the participants. By plotting only the samples of imputed GSA-MD and ulcWGS data (Supplementary Figure S4), we observed some spatial distance between identical samples generated by these two platforms. We computed kinship coefficients using all of the ancestry-informative markers to quantitatively capture genomic distance and similarity between these samples. Pairwise kinship coefficients among identical samples generated by GSA-MD and ulcWGS ranged from 0.45 to 0.49, indicating minor to no discrepancy in ancestry prediction between the two platforms. Similarly, we also estimated Pearson R^2^ correlation of the 10 PC scores and observed strong correlations (R^2^ > 0.95) between numerical PC values of samples generated by ulcWGS and GSA-MD platforms (Supplementary Figure S5). We further compared the performance of ulcWGS vs. GSA-MD in refining EUR population substructure. With EUR 1 KGP phase 3 and AJ population as reference, we performed PCA and plotted the top 2 PCs (Figure 5B). Both ulcWGS and GSA-MD were able to comparably predict EUR population substructure, as exampled by the three labeled pair sample duplicates from these two platforms overlaid on top of the distinct EUR subclusters (sample 03 on CEU; sample 30 on TSI; and sample 55 on AJ).

## Discussion

To date, genotyping-based approach using SNP arrays has been a standard practice in GWAS designs. In this report, we explored, for the first time, the feasibility and effectiveness of ulcWGS (0.4× coverage) as a sequencing-based GWAS alternative to standard SNP array genotyping. Currently, there were only a handful of studies assessing the capacity of ulcWGS (0.1–0.5×) in GWAS-based strategies (Pasaniuc et al., 2012; Homburger et al., 2019; Wasik et al., 2021). Those prior reports primarily have not stemmed from actual sequencing, but *in silico* methods of simulation (Pasaniuc et al., 2012) or downsampling (Homburger et al., 2019; Wasik et al., 2021). These studies reported high imputation accuracy (R^2^ > 0.90) and genotype concordance with consistent effect estimates and association *p*-values of complex disease risk stratification using single-variant or genome-wide polygenic score (GPS) approaches, compared with prior genotyping-based GWAS designs. However, given the lack of real-experiment ulcWGS, these conclusions could be prone to biases. While simulation-based study suggested that ulcWGS with a target coverage as low as 0.1× may be of utility in GWAS (Pasaniuc et al., 2012), there has only been one recent report using actual low-pass sequencing experimental design (0.5× ulcWGS) (Li et al., 2021). By direct whole-genome sequencing of 60 European individuals to a coverage of 0.5–1×, this prior report (Li et al., 2021) showed that ulcWGS-based GWAS approach delivered increased genotyping accuracy (0.90 non-reference concordance for ulcWGS vs. 0.83 for Illumina GSA array) and improved polygenic risk score estimation for complex traits, making it a promising alternative to an array-based approach.

In contrast to prior reports, our study performed direct sequencing to less than 0.5× depth, which significantly improves the cost compared with that of the popular GSA-MD. In addition, we were also able to assess the performance of ulcWGS using hcWGS (30×) as a gold standard, providing another layer of evidence in support of ulcWGS, compared with GSA-MD SNP arrays. Also, in our analysis, we adopted the most recent bioinformatics tools for ulcWGS data shown to outperform previous imputation pipelines in terms of both computational time and accuracy across allele frequency spectrum (Rubinacci et al., 2021). In summary, our data showed that ulcWGS captured comparable proportion of genetic variation with imputed data from GSA-MD array and importantly captured higher fraction of rare variants (MAF <1%) from 30× hcWGS platform. Also, ulcWGS performed better than GSA-MD in terms of imputation R^2^ score and genotype accuracy, particularly for low and common variants (MAF >1%). Our study also highlighted the effectiveness of ulcWGS in sex imputation and ancestry prediction. The ulcWGS correctly assigned the participants by sex, and all the participants were clustered by the self-reported European ancestry, with the high correlation for the top 10 PC scores in each sample duplicate. In addition, our analysis also showed that ulcWGS performs with the same accuracy as GSA-MD chip in predicting European population substructure, which is reassuring as GSA-MD chip is highly curated for ancestry-informative markers (AIMs) of European ancestry. The highly comparable performance of both platforms observed in our data further supports the use of ulcWGS in population genetic scans.

These findings strongly suggest that ulcWGS with sequencing depth of as low as 0.4× is sufficient to generate information that is not only comparable with GSA-MD arrays but also supersedes the accuracy of SNP array genotyping, particularly in imputation score accuracy and concordance. This further strengthens its use in GWAS design, given several practical advantages over genotyping-based arrays. The current cost of 0.4× WGS per sample, including library preparation, was approximately $30 ($12 for sequencing and $18 for library preparation per sample), lower than the cost of GSA array ($39 per sample). While the budget saved per sample appears negligible, it quickly adds up to a considerable magnitude in large-scale population GWAS designs. In addition, the 0.4X ulcWGS platform requires remarkably less amount of DNA input (10–20 ng) compared with genotyping arrays (40–200 ng), making it even more attractive alternative especially considering clinically precious or compromised DNA material. Of note, while the low-DNA input in ulcWGS requires PCR pre-amplification (eight cycles in our study), which may introduce potential bias, the comparable results observed between 30× hcWGS, GSA-MD, and ulcWGS in our data strongly suggest that such potential bias is minimal.

The study has certain limitations. While we only assessed the efficacy of ulcWGS among participants of European descent limiting the conclusions to other ancestries, we predict that ulcWGS performs similarly or better in other populations, particularly those of high genetic diversity such as African populations. As highlighted in recent reports (Martin et al., 2021; Li et al., 2021), lcWGS (0.5–1×) outperformed genotyping arrays in African populations, particularly for the detection of rare variants (MAF <1%). Our study was also limited to a comparison of ulcWGS against only one type of genotyping array (GSA-MD); however, GSA-MD is one of the most commonly used current array for cost-effective and comprehensive genome-wide scan of multi-ethnic populations. We were also unable to fully estimate true positives/positive predictive values (PPV) for novel and rare variants (MAF <1%) uniquely captured by ulcWGS, due to a relatively small sample size of our study.

In conclusion, despite the limitations of the study, this report underscores the significance of ulcWGS (<0.4×) as a competitive alternative to genotyping arrays in next-generation GWAS, given its comparable accuracy, affordable cost, and low-DNA input needed. Future studies are warranted to evaluate the power of ulcWGS platform in replicating or discovering novel GWAS signals exerted by the effects of rare variation, in complex trait association analyses in European and other ancestries.

## Data Availability

The datasets presented in this article are not readily available due to patient privacy and IRB. Requests to access the datasets should be directed to the authors.
