# Supplementary material for: Ultra Low-Coverage Whole-Genome Sequencing as an Alternative to Genotyping Arrays in Genome-Wide Association Studies
Source: Front Genet. 2022 Feb 15;12:790445. doi: 10.3389/fgene.2021.790445 (PMC8889143; doi:10.3389/fgene.2021.790445)
Supplement: Supplementary file 1 [file DataSheet1.docx]

**Supplementary**

**
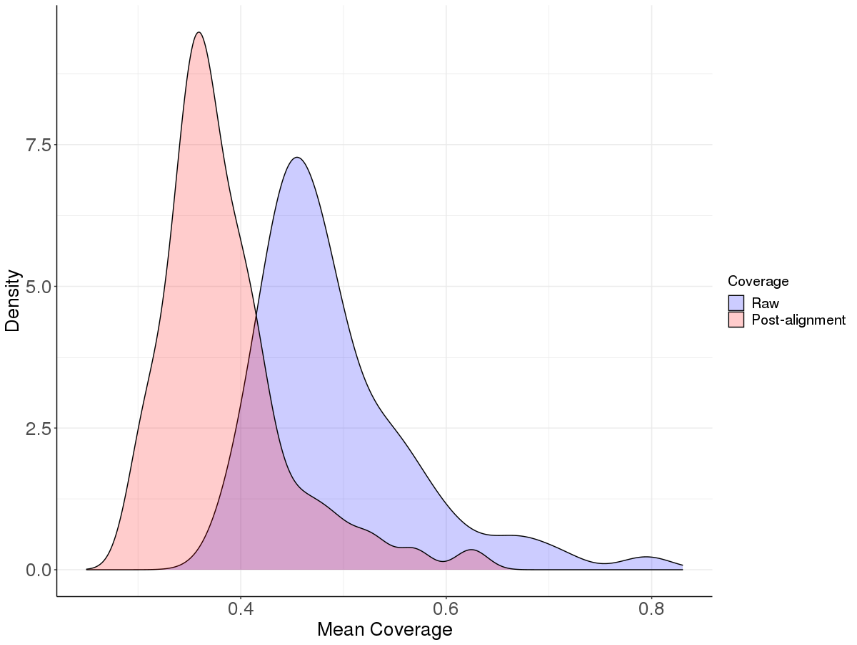
**

| **R^2^ thresholds** | **Indels** | |  | **SNPs** | |
| --- | --- | --- | --- | --- | --- |
|  | Imputed GSA-MD | ulcWGS |  | Imputed GSA-MD | ulcWGS |
| 0.9 | 501,560 | 677,631 |  | 5,347,851 | 6,738,203 |
| 0.8 | 783,314 | 883,154 |  | 7,344,231 | 8,029,180 |
| 0.6 | 1,069,374 | 1,106,649 |  | 9,033,161 | 9,353,873 |
| 0.5 | 1,150,114 | 1,177,922 |  | 9,506,122 | 9,809,345 |
| 0.3 | 1,237,400 | 1,263,160 |  | 10,009,322 | 10,319,730 |
| Unfiltered R^2^ | 1,264,727 | 1,280,217 |  | 10,157,732 | 10,384,933 |

**Figure S1**: **Distributions of raw sequencing mean coverage vs. post-alignment coverage for ulcWGS data**. We estimated the raw sequencing coverage for each sample by dividing number of read yields (Mbases) by the whole genome size (~3.3Gb). The overall mean sequencing coverage was 0.49X. After alignment by BWA-mem to the human genome build GRCh37, we calculated post-alignment sequencing coverage excluding non-primary alignments, duplicate and unmapped reads. The post-alignment mean sequencing coverage for all samples was 0.38X, similar to our target coverage of 0.4X.

**Table S1: Non-monomorphic variants captured by ulcWGS and imputed GSA-MD at different imputation R^2^ thresholds.** Non-monomorphic indels and SNPs from both imputed GSA-MD and ulcWGS platforms were filtered at multiple imputation R^2^ thresholds (R^2^>0.3; >0.5; >0.6; >0.8; and >0.9). We consistently observed similar number of both indels and SNPs from ulcWGS in comparison with imputed GSA-MD.

| **Population MAF** | **Total 30X hcWGS non-monomorphs** |  | **Fraction of 30X hcWGS non-monomorphs recapitulated by:** | |
| --- | --- | --- | --- | --- |
|  |  |  | Imputed GSA-MD (N; %) | UlcWGS (N; %) |
| MAF <1 | 958,802 |  | 380,098 (39.64) | 445,639 (46.48) |
| MAF:1-5 | 1,202,781 |  | 947,275 (78.75) | 958,041 (79.65) |
| MAF>5 | 6,420,887 |  | 5,640,633 (87.84) | 5,666,390 (88.24) |

**Table S2: Number of variants from 30X hcWGS recapitulated by ulcWGS and imputed GSA-MD.** For a subset of N=13 participants with data from all three platforms (30X hcWGS, imputed GSA-MD, and ulcWGS), we filtered to retain only non-monomorphic variants, and assessed the comprehensiveness of imputed GSA-MD and ulcWGS in capturing 30X hcWGS variants. We showed here the number of total variants from 30X hcWGS at each population MAF bins, and estimated the fractions of those variants captured by imputed GSA-MD vs ulcWGS.

| **Population MAF** |  | **Indels** | |  | **SNPs** | |
| --- | --- | --- | --- | --- | --- | --- |
|  |  | Imputed GSA-MD | ulcWGS |  | Imputed GSA-MD | ulcWGS |
| MAF <1 |  | 0.08 | 0.09 |  | 0.06 | 0.05 |
| MAF:1-5 |  | 0.66 | 0.68 |  | 0.72 | 0.72 |
| MAF>5 |  | 0.81 | 0.86 |  | 0.87 | 0.93 |

**Table S3: Mean imputation R^2^ scores.** We estimated mean imputation R^2^ of ulcWGS and imputed GSA-MD stratified by population MAF (MAF<1% rare, MAF:1-5% low frequency and MAF > 5% common).

| **Imputation R^2^ threshold** | **Indels** | | | |  | **SNPs** | | | |
| --- | --- | --- | --- | --- | --- | --- | --- | --- | --- |
|  | MAF <1 | MAF:1-5 | MAF>5 | Overall |  | MAF <1 | MAF:1-5 | MAF>5 | Overall |
| 0.3  0.5 | 0.60  0.68 | 0.76  0.81 | 0.87  0.88 | 0.82  0.84 |  | 0.60  0.67 | 0.78  0.81 | 0.91  0.91 | 0.82  0.85 |
| 0.6 | 0.73 | 0.83 | 0.89 | 0.86 |  | 0.71 | 0.83 | 0.92 | 0.87 |
| 0.8 | 0.82 | 0.90 | 0.93 | 0.91 |  | 0.80 | 0.87 | 0.94 | 0.91 |
| 0.9 | 0.88 | 0.93 | 0.95 | 0.94 |  | 0.85 | 0.90 | 0.95 | 0.93 |

**Table S4: Mean non-reference concordance of unfiltered ulcWGS data and imputed gold-standard GSA-MD, filtered at respective** R^2^ **thresholds.** The overall mean non-reference concordance for ulcWGS data was estimated separately for indels and SNPs using the imputed GSA-MD variants filtered at various imputation R^2^ thresholds as gold-standard.

| **Imputation R^2^ threshold** | **Indels** | | | |  | **SNPs** | | | |
| --- | --- | --- | --- | --- | --- | --- | --- | --- | --- |
|  | MAF <1 | MAF:1-5 | MAF>5 | Overall |  | MAF <1 | MAF:1-5 | MAF>5 | Overall |
| 0.3 | 0.77 | 0.80 | 0.87 | 0.85 |  | 0.78 | 0.82 | 0.91 | 0.87 |
| 0.5 | 0.88 | 0.86 | 0.89 | 0.88 |  | 0.89 | 0.86 | 0.92 | 0.90 |
| 0.6 | 0.91 | 0.88 | 0.90 | 0.90 |  | 0.91 | 0.88 | 0.92 | 0.92 |
| 0.8 | 0.96 | 0.94 | 0.94 | 0.94 |  | 0.96 | 0.94 | 0.95 | 0.95 |
| 0.9 | 0.98 | 0.97 | 0.97 | 0.97 |  | 0.98 | 0.97 | 0.97 | 0.97 |

**Table S5: Mean non-reference concordance of ulcWGS data and imputed gold-standard GSA-MD, both filtered at respective R^2^ thresholds.** We estimated overall mean non-reference concordance of ulcWGS against the imputed GSA-MD, for both indels and SNPs. Both data were filtered at different imputation R^2^ thresholds as shown.

| **Population MAF** |  | **Indels** | |  | **SNPs** | |
| --- | --- | --- | --- | --- | --- | --- |
|  |  | Imputed GSA-MD | ulcWGS |  | Imputed GSA-MD | ulcWGS |
| MAF <1 |  | 0.64 | 0.74 |  | 0.66 | 0.70 |
| MAF:1-5 |  | 0.76 | 0.79 |  | 0.83 | 0.84 |
| MAF>5 |  | 0.87 | 0.88 |  | 0.93 | 0.94 |
| Overall |  | 0.83 | 0.86 |  | 0.88 | 0.90 |

**Table S6: Mean non-reference concordance of ulcWGS and imputed GSA-MD using 30X hcWGS as gold-standard.** We summarized here mean non-reference concordance of ulcWGS and imputed GSA-MD stratified by population MAF (MAF<1% rare, MAF:1-5% low frequency and MAF > 5% common).


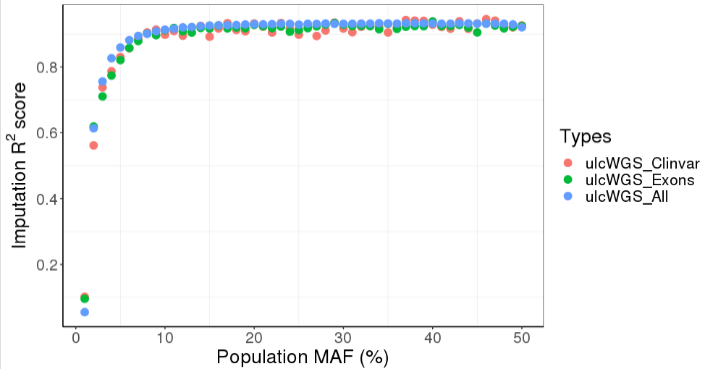

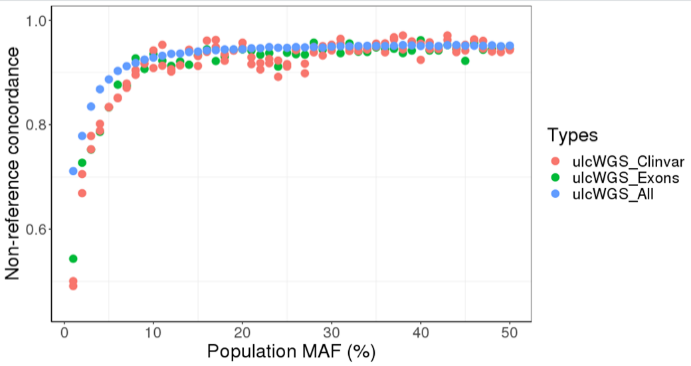


**A.**

**B.**

**Figure S2**: **Imputation R^2^ score and non-reference concordance for Clinvar, exonic and all variants generated by ulcWGS platform.** We assessed the robustness of ulcWGS platform in the accurate capturing of clinically important variants assayed on the GSA-MD chip (i.e clinvar and exonic variants). Using the typed GSA-MD genotypes as the “truth”, we observed comparable imputation accuracy (Panel **A**), and non-reference concordance (Panel **B**) among all variants in comparison to clinvar and exonic variants. Note: Mean non-reference concordance estimates in Panel B appeared less stable for clinvar and exonic variants, as reflected by the wavy curves, partly due to the limited number of variants in each bin as a result of the exclusion of homozygote reference concordance.


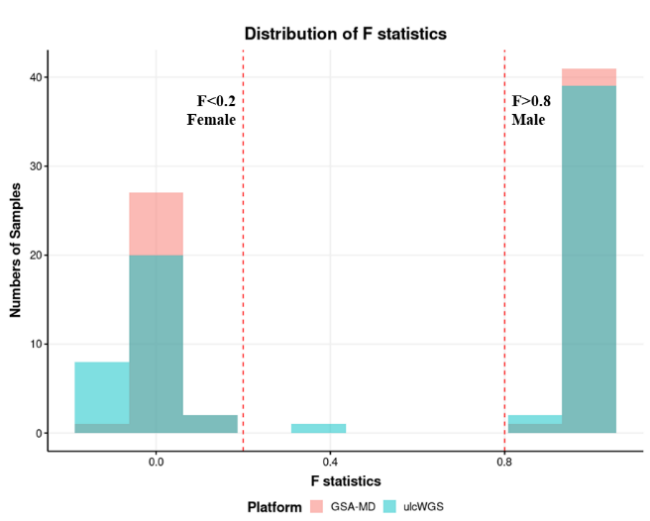


**Figure S3: Distribution of chromosome X inbreeding F-statistics from ulcWGS and GSA-MD chip.** We observed two tight clumps of F estimates for females (left clump: F<0.2) and for males (right clump: F>0.8) from both platforms (light pink: GSA-MD, and light blue: ulcWGS). One sample from ulcWGS, was not assigned for sex with F-estimate falling between the male and female thresholds (F=0.37).


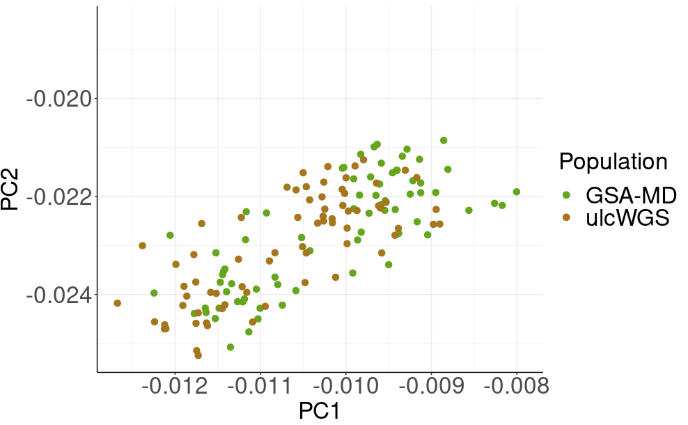


**Figure S4: A zoom-in scatterplot of population stratification for the study cohort from GSA-MD and ulcWGS.** From the main Figure 5A, we zoomed in and plotted the top 2 PC scores only for our study population to further investigate distance between identical samples generated by ulcWGS vs. imputed GSA-MD data.

**
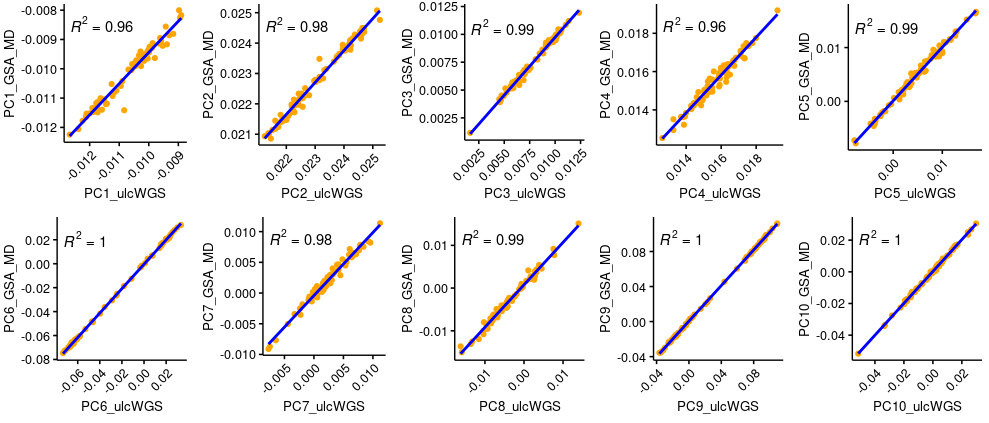
Figure S5: Pearson R^2^ coefficient of determination for top 10 PC scores generated by ulcWGS vs GSA-MD chip.** Values on the X-axis and Y-axis represented PC scores from ulcWGS, and GSA-MD chip, respectively. Each dot plotted a data point of each study participant. Blue lines represent smooth regression lines of the two platforms. We observed high correlation of the top 10 PC scores (R^2^>0.95) generated by ulcWGS and GSA-MD.
